# Untargeted Metabolomics Reveals Distinct Serum Metabolic Profiles in Avian Influenza Occupational Exposure Populations

**DOI:** 10.3390/metabo15100663

**Published:** 2025-10-11

**Authors:** Shuoqin Mao, Lei Wang, Jing Su, Caihua Long, Muti Mahe, Zhenguo Gao, Jia Liu

**Affiliations:** 1 School of Basic Medical Sciences, Capital Medical University, Beijing 100069, China; 2 National Key Laboratory of Intelligent Tracking and Forecasting for Infectious Diseases, National Institute for Communicable Disease Control and Prevention, Chinese Center for Disease Control and Prevention, Beijing 102206, China; 3 Xinjiang Uygur Autonomous Region Center for Disease Control and Prevention, Xinjiang Key Laboratory of Vector-borne Infectious Diseases, Urumqi 830000, China; 4 Department of Microbiology and Infectious Disease Center, School of Basic Medical Sciences, Peking University Health Science Center, Beijing 100191, China

**Keywords:** untargeted metabolomics, lipidomics, avian influenza, occupational exposure, LC-MS

## Abstract

Background and Objectives: Avian influenza poses a continuous public health threat, particularly to individuals with occupational exposure to poultry such as farm workers, live animal market employees, and processing plant staff. This study aimed to investigate the systemic metabolic effects of such exposure and to identify potential biomarkers for early detection and health risk assessment. Materials and Methods: An untargeted liquid chromatography–mass spectrometry (LC-MS)-based metabolomics approach was applied to analyze serum samples from occupationally exposed individuals and healthy controls. Multivariate statistical analysis, pathway enrichment, and topology analysis were performed to identify significantly altered metabolites and metabolic pathways. The least absolute shrinkage and selection operator (LASSO) algorithm was employed to select key metabolites. Results: Multivariate statistical analysis revealed a clear separation between the exposure group and control, suggesting distinct metabolic profiles between the two populations. Pathway analysis indicated significant alterations in alanine, aspartate, and glutamate metabolism, as well as tryptophan metabolism, which are closely linked to immune regulation, energy metabolism, and host–pathogen interactions. LASSO feature selection and subsequent manual verification identified 17 key metabolites with strong discriminative power. Furthermore, lipidomic profiling revealed a pronounced increase in lysophosphatidylcholine (LPC) levels and a concurrent decrease in phosphatidylcholine (PC) species in exposed individuals. Conclusions: This study reveals metabolic disruptions associated with occupational avian influenza exposure and identifies potential serum biomarkers related to immune and lipid metabolism. These findings provide novel insights into host responses to avian influenza exposure and may support early detection and health risk assessment in high-risk occupational populations.

## 1. Introduction

Avian influenza, commonly known as bird flu, is caused by influenza A viruses and poses a significant public health concern due to its zoonotic potential [1]. Although human infections are relatively rare, highly pathogenic strains such as H5N1 and H7N9 have been associated with severe disease and high mortality [2,3]. Occupational exposure represents an important risk factor, particularly for individuals working in poultry farming, processing, and veterinary services, where frequent contact with infected birds or contaminated environments can facilitate transmission [4]. Notably, even in the absence of clinical symptoms, such exposure may induce subtle immune alterations and inflammatory responses, raising concerns about its broader physiological impact. Given that metabolic alterations often precede clinical symptoms, studying the metabolic profiles of exposed workers may reveal exposure-related biochemical changes and provide insights into subclinical physiological effects. Furthermore, understanding how prolonged exposure to avian influenza affects metabolic pathways could inform the development of preventive strategies and therapeutic approaches for those at highest risk, while also contributing to broader public health efforts to control zoonotic transmission.

Metabolomics is a powerful analytical approach that provides a comprehensive profile of small molecule metabolites in biological samples [5]. By characterizing metabolites that reflect dynamic biochemical processes, untargeted metabolomics can reveal early metabolic disturbances before clinical symptoms appear [6]. Metabolomics thus offers the potential to identify early biomarkers of infection, monitor disease progression, and understand the metabolic disruptions induced by viral exposure, providing valuable insights for public health surveillance and intervention strategies against avian influenza.

In this study, we employed untargeted metabolomics based on liquid chromatography–mass spectrometry (LC-MS) to investigate the differences in the serum metabolomes of individuals with occupational exposure to avian influenza and healthy controls. By analyzing the metabolic profiles of both groups using least absolute shrinkage and selection operator (LASSO), we successfully identified key metabolites that are altered in response to avian influenza exposure. This approach not only contributes to a deeper understanding of the biochemical changes induced by avian influenza but also holds the potential to identify early biomarkers for detection, improve risk assessments, and design preventive strategies for those at high occupational risk.

## 2. Experimental Section

### 2.1. Study Subjects and Inclusion Criteria

The occupationally exposed group comprised individuals working in high-risk environments associated with poultry, including large-scale poultry farms, live poultry wholesale markets, and poultry slaughter and processing plants. These workers were exposed to avian influenza through various activities, such as feeding, capturing, cleaning poultry houses, and processing poultry. The healthy control group consisted of individuals undergoing routine health check-ups, with no history of occupational exposure to avian influenza or related viruses. Both male and female participants were included to minimize gender bias. Individuals were excluded based on the following criteria: (1) a history of major infectious diseases (e.g., HIV, HBV, HCV, tuberculosis), metabolic disorders (e.g., diabetes mellitus, thyroid dysfunction), or autoimmune conditions; (2) a history of severe influenza or respiratory illnesses within the past 6 months; (3) pregnancy or lactation; (4) heavy alcohol consumption (defined as >30 g/day for men or >20 g/day for women); or (5) heavy smoking (defined as >10 cigarettes per day). These exclusions were applied to minimize potential confounders of the serum metabolic profile.

The study was approved by the Ethical Committee of the National Institute for Communicable Disease Control and Prevention, Chinese Center for Disease Control and Prevention with Approval No.: ICDC-LPJ-2024008.

### 2.2. Sample Pretreatment

A volume of 100 μL of serum sample was thawed at 4 °C or on ice for approximately 2 h. Prior to extraction, Eppendorf (EP) tubes were pre-cooled to maintain low-temperature conditions. For global metabolite extraction, which targets both endogenous and exogenous small molecule metabolites, 400 μL pre-chilled methanol was added to each sample. The mixture was vortexed thoroughly for 10 min and then incubated at −80 °C for 20 min to enhance protein precipitation efficiency. Samples were then centrifuged at 14,000 rpm for 10 min at 4 °C, and the resulting supernatants were recovered and dried under vacuum via rotary evaporation at 4 °C. For quality control (QC), 20 μL of each sample was pooled, mixed thoroughly, and aliquoted into 100 μL portions to assess system stability and reproducibility.

### 2.3. Instrument Analysis

Untargeted metabolomic analysis was performed using a Vanquish plus ultra-high performance liquid chromatography (UHPLC) system (Thermo Fisher Scientific, Germering, Germany) coupled with a Q Exactive Plus Orbitrap mass spectrometer (Thermo Fisher Scientific, Bremen, Germany). Chromatographic separation was achieved using a Thermo Acclaim Rapid Separation Liquid Chromatography (RSLC) 120 C18 column (100 × 2.1 mm, 2.2 µm) maintained at 40 °C, with mobile phase A consisting of 0.1% formic acid in water and mobile phase B consisting of 0.1% formic acid in acetonitrile. The flow rate was set at 0.35 mL/min. For positive ion mode, a gradient program was used as follows: 2% B at 1.0 min, increasing to 98% B at 16.0 min, maintained at 98% B until 20.0 min, and then returned to 2% B at 22.0 min. For negative ion mode, the gradient was: 2% B at 2.0 min, increasing to 98% B at 17.0 min, maintained at 98% B until 22.0 min, and then returned to 2% B at 24.0 min [7]. The MS system was operated in both positive and negative ion mode with top10 acquisition strategies. The full MS scan was acquired with a resolution of 70,000, an automatic gain control (AGC) target of 1 × 10^6^, a maximum ion injection time (IT) of 100 ms, and a scan range of 70~1050 *m*/*z*. Data-dependent MS^2^ (dd-MS^2^) analysis was conducted using a top10 method with a resolution of 17,500, an AGC target of 2 × 10^5^, a maximum IT of 50 ms, an isolation window of 1.6 *m*/*z*, and stepped normalized collision energies (NCE) of 20, 40, and 60 eV.

### 2.4. Data Analysis and Mining

The acquired raw data were processed using MS-DIAL [8] for peak detection, alignment, and annotation of metabolites. Pathway analysis and enrichment analysis were performed in MetaboAnalyst [9]. To identify significantly altered lipids, the Wilcoxon rank-sum test was applied, followed by Benjamini–Hochberg correction. Lipids with adjusted *p* < 0.05 and fold change >2 or <0.5 were considered significant. Principal component analysis (PCA), stacked bar chart and bubble plot were conducted using R (version 4.3.1) with ggplot2 package. Package glmnet was applied for LASSO regression to reduce the number of variables by identifying the most significant metabolites associated with bird flu exposure.

## 3. Results and Discussion

### 3.1. Clinical Characteristic of Subjects

A total of 177 occupationally exposed workers and 59 healthy control subjects were enrolled in this study. The main two exposure modes were processing activities (slaughtering, defeathering, etc.) (49.7%) and poultry-related tasks (feeding, cleaning poultry houses, capturing birds) (41.2%). Environmental samples from their workplaces were collected and tested using real time polymerase chain reaction (RT-PCR), and the results confirmed their exposure to avian influenza viruses (e.g., H5 and H9 subtypes), see Table S1 in Supplementary Material for detail. The age and gender were matched between occupationally exposed group and healthy controls to minimize confounding factors that might influence serum metabolite profiles. The clinical characteristics are detailed in Table 1.

### 3.2. Metabolomic Analysis

**Figure 1:**
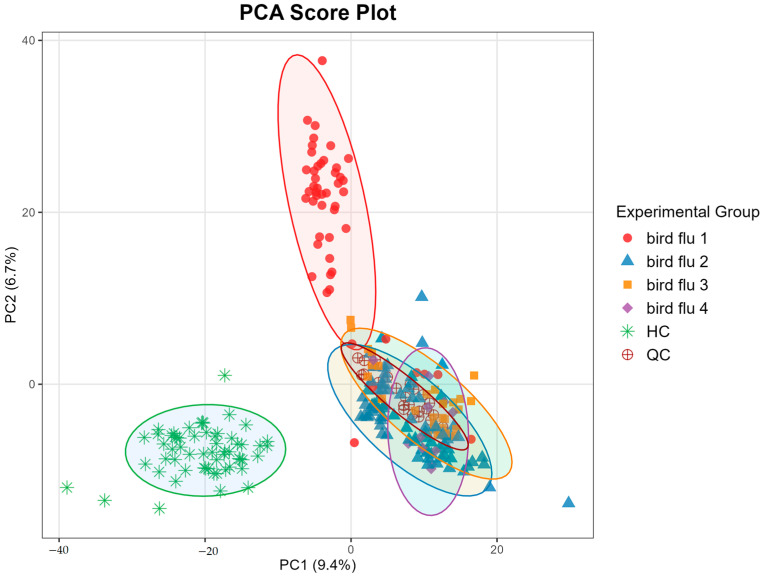
PCA score plots of identified molecular features in negative ion mode. HC, healthy control; QC, quality control; bird flu 1, large-scale poultry farms; bird flu 2, live poultry wholesale markets; bird flu 3, poultry slaughter and processing plants; bird flu 4, backyard poultry farmers.

To assess the overall metabolic differences between groups and evaluate data quality, PCA was performed on the serum metabolomic profiles. As shown in the PCA score plot (Figure 1), QC samples clustered tightly near the center of the plot, indicating high instrumental stability and good reproducibility throughout the acquisition process. In contrast, samples from the occupationally exposed group (bird flu) and the healthy control group (HC) were clearly separated along the principal components, suggesting distinct metabolic profiles between the two populations. Notably, the subgroup from large-scale poultry farms formed a distinct cluster, separate from both other exposed subgroups and healthy controls. This observation reflects a unique metabolic profile associated with this specific occupational environment, which warrants further investigation into the specific pathways and metabolites involved.

### 3.3. Enrichment and Pathway Analysis

**Figure 2:**
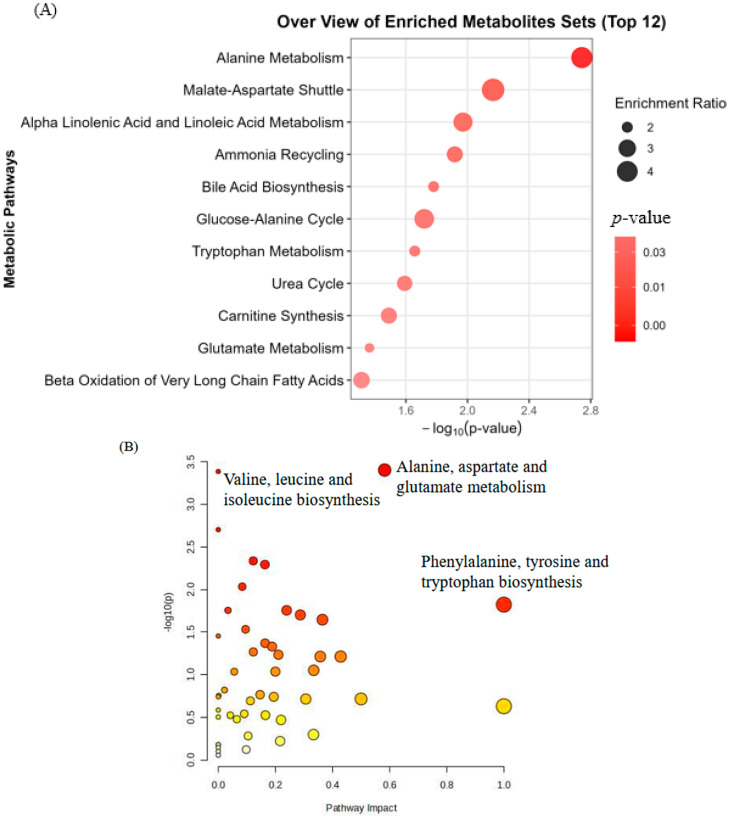
Enrichment analysis (**A**) and pathway analysis (**B**) of the Avian Influenza-Exposed Individuals. Alanine metabolism was the most significantly disturbed pathway in both enrichment and topology analyses.

Metabolites were considered significantly altered if they met the criteria of a fold change >2 or <0.5 and a *p* value < 0.05. See Figure S1 for a volcano plot showing these significant metabolites. Enrichment analysis revealed several significantly enriched metabolic pathways, reflecting systemic biochemical alterations potentially associated with avian influenza exposure, see Figure 2A. Notably, alanine metabolism (*p* = 0.00181), malate-aspartate shuttle (*p* = 0.00682), and alpha linolenic acid and linoleic acid metabolism (*p* = 0.0107) were among the top enriched pathways, suggesting disruptions in amino acid metabolism and lipid signaling in exposed individuals. Additionally, pathways such as ammonia recycling, bile acid biosynthesis, and the glucose-alanine cycle were also significantly enriched, indicating possible alterations in nitrogen metabolism, hepatic function, and energy balance.

Following enrichment analysis, pathway topology analysis was performed to further explore the biological relevance and systemic impact of the differential metabolites (Figure 2B). Among the significantly perturbed pathways, alanine, aspartate, and glutamate metabolism emerged as the most prominently affected (*p* = 0.00039). This pathway plays a central role in nitrogen balance, neurotransmitter cycling, and energy metabolism [10], and its disruption may reflect immune activation and mitochondrial stress in response to viral antigens [11]. Similarly, valine, leucine and isoleucine biosynthesis (*p* = 0.00041) also showed strong statistical significance, indicating perturbations in branched-chain amino acid metabolism, which are closely linked to inflammation [12], muscle metabolism, and immune cell signaling [13]. Other significantly affected pathways included biosynthesis of unsaturated fatty acids, arginine biosynthesis, and glyoxylate and dicarboxylate metabolism.

### 3.4. Biological Pathway Insights

Two pathways were notably altered: alanine, aspartate and glutamate metabolism was the most significantly disturbed pathway in both enrichment and topology analyses (*p* < 0.05 for both), and tryptophan metabolism was significantly altered in the enrichment analysis while showing a strong trend toward significance (*p* = 0.058) in the topology analysis.

Alanine, aspartate and glutamate metabolism pathway is central to multiple physiological processes, including transamination reactions, nitrogen shuttling, neurotransmission [14], and mitochondrial energy metabolism. Glutamate and aspartate serve not only as amino acid substrates but also as metabolic intermediates that link carbon and nitrogen metabolism [15]. This pathway could undergo significant remodeling facing viral infection. For example, glutamine serves as a key substrate for oxidative metabolism in activated T cells and macrophages [16], while aspartate supports nucleotide biosynthesis and the malate-aspartate shuttle, which maintains mitochondrial nicotinamide adenine dinucleotide (NAD+/NADH) balance [17]. The observed dysregulation of this pathway in occupationally exposed individuals may reflect several converging mechanisms: (1) increased demand for biosynthetic precursors to support activated immune cells; (2) enhanced glutaminolysis and anaplerosis to fuel the tricarboxylic acid (TCA) cycle under inflammatory conditions; and (3) oxidative stress-induced mitochondrial dysfunction leading to metabolic alterations. Importantly, previous studies have shown that influenza virus can manipulate host amino acid metabolism to facilitate replication and immune evasion [18]. Thus, alterations in alanine, aspartate, and glutamate metabolism may represent a host metabolic adaptation to persistent or repeated low-level exposure to avian influenza antigens in occupational settings.

Tryptophan metabolism is a well-established immune-regulatory pathway, primarily mediated by the indoleamine 2,3-dioxygenase (IDO) and tryptophan 2,3-dioxygenase (TDO) enzymes [19]. Upon activation by pro-inflammatory cytokines such as interferon-γ (IFN-γ), IDO catalyzes the conversion of tryptophan to kynurenine, a metabolite that plays dual roles in immunosuppression and neuroinflammation [20]. Elevated kynurenine levels have been associated with immune tolerance, T-cell exhaustion, and regulation of macrophage phenotypes-mechanisms that may limit immunopathology but also contribute to viral persistence. Persistent low-dose influenza virus stimulation may chronically activate the IDO pathway, leading to sustained tryptophan depletion and accumulation of downstream immunosuppressive metabolites. This metabolic shift could represent a compensatory anti-inflammatory response aimed at avoiding tissue damage from excessive inflammation.

### 3.5. Key Metabolites Identification by Machine Learning

**Figure 3:**
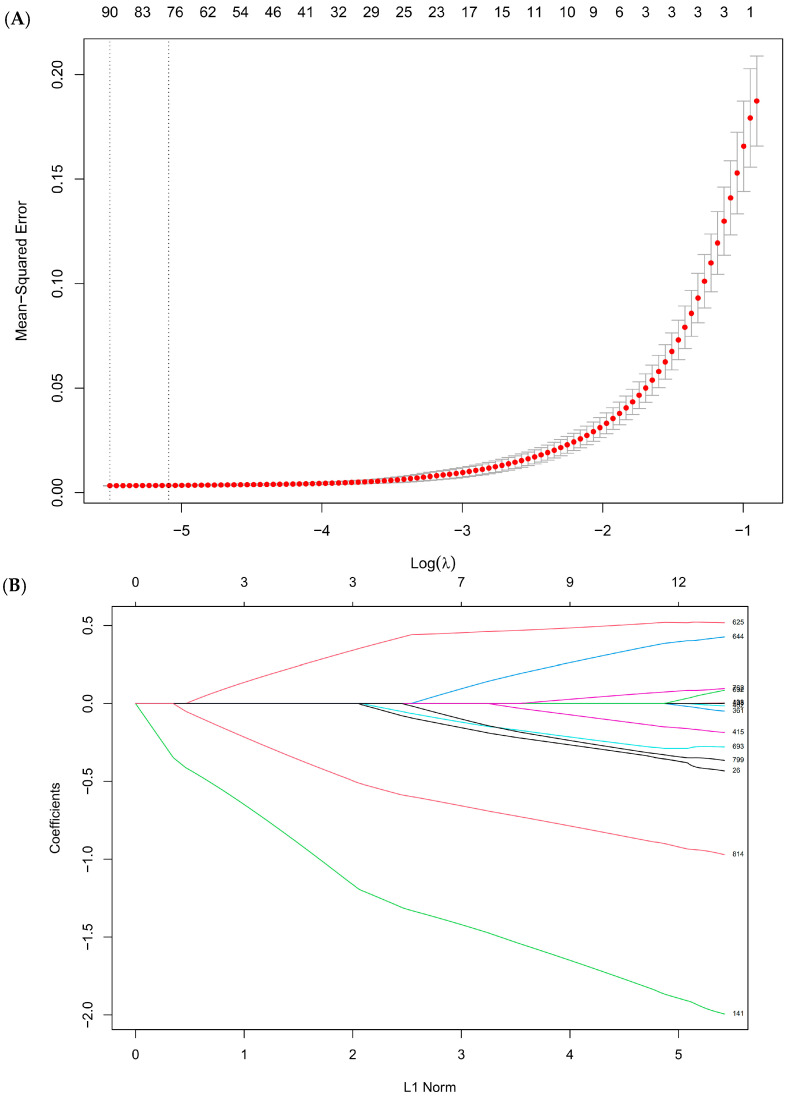
(**A**) Cross-validation curve in LASSO regression. The x-axis represents the log value of the penalty parameter (lambda, λ), which controls the strength of regularization. The y-axis represents mean-squared error. (**B**) Coefficient path plot illustrates the trajectory of metabolite coefficients as the penalty parameter decreases.

To further refine the set of potential biomarkers and identify key metabolites associated with occupational exposure to avian influenza, we applied LASSO regression algorithm to the pool of differential metabolites identified in Section 3.3 (fold change >2 or <0.5, *p* < 0.05). Figure 3 illustrates the cross-validation curve and coefficient path plot in LASSO regression. The cross-validation curve indicates the mean-squared error for different values of the tuning parameter λ, with error bars representing standard errors. The coefficient path plot illustrates the trajectory of metabolite coefficients as the penalty parameter decreases. We selected λ_min, which corresponds to the minimum mean-squared error. As a result, a total of 90 metabolites were retained as candidates. Then, we excluded compounds with ambiguous or unreliable annotations, such as drug-related molecules or xenobiotics that may arise from environmental sources rather than biological processes. Chromatographic peak shapes and tandem mass spectrometry (MS/MS) spectra of the remaining candidates were carefully examined with manual curation to identify each metabolite. Finally, 17 key metabolites were retained as high-confidence biomarkers associated with avian influenza occupational exposure, as listed in Table 2.

**Figure 4:**
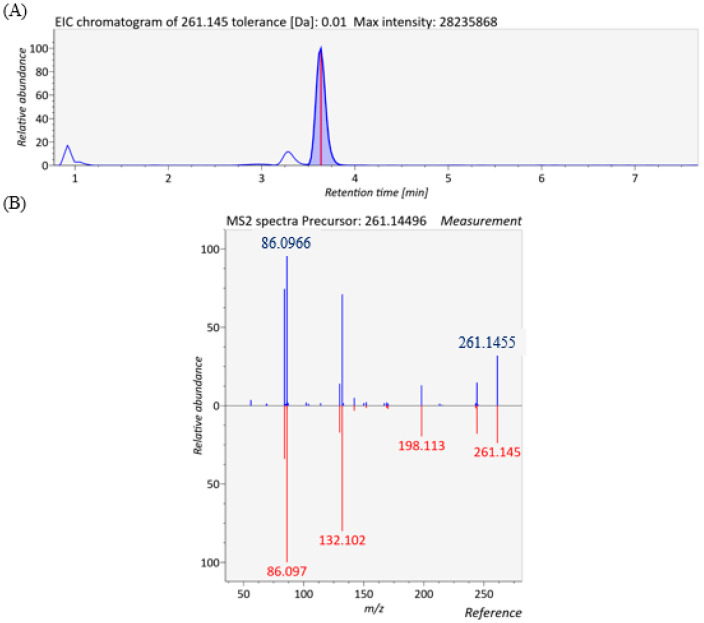
EIC chromatogram (**A**) and MS/MS spectra (**B**) of glutamylleucine.

9-hydroxy-octadecadienoic acid (9-HODE), a lipid peroxidation product derived from linoleic acid, was found to be significantly decreased in the exposed group. This finding is consistent with previous studies. A metabolomic study in mice showed that plasma 9-HODE levels declined markedly after influenza A virus (IAV) infection [21]. Similarly, in vitro experiments using human bronchial epithelial cells (16HBE) infected with IAV also demonstrated a significant reduction in 9-HODE concentrations [22]. We also observed significantly increased xanthine and hypoxanthine, which is in line with a very recent study. Shi et al. reported serum concentrations of xanthine and hypoxanthine were significantly elevated in children following IAV infection [23]. Glutamine was up-regulated in the avian-influenza-exposed group in our study. This aligns with prior findings demonstrating that IAV infection markedly increases glutamine levels in A549 cells, suggesting virus-driven perturbations of host nucleotide metabolism [24]. In addition, the glutamate metabolism pathway was disturbed as discussed in Section 3.4. Lauroylcarnitine, a medium- to long-chain acylcarnitine, was significantly increased in the exposed group. These metabolite levels were also found to increase with Corona Virus Disease 2019 (COVID-19) severity, reaching their highest abundance in patients with severe and fatal outcomes [25]. In addition, glutamylleucine, androstane-3,17-diol, Sphinganine 1-phosphate, glycochenodeoxycholate and 3-indoleacetic acid were found to be significantly changed in the exposed group. The extracted ion chromatogram (EIC) and MS/MS spectrum of glutamylleucine are shown in Figure 4, with a dot product similarity score 926.3351. See Figure S2 for representative spectra for several other key metabolites.

### 3.6. Lipidomic Analysis

Given that sample preparation in this study was based on protein precipitation using cold methanol, it is expected that a broad spectrum of small molecules, including both polar metabolites and lipids, were efficiently extracted and retained for mass spectrometric detection. To specifically characterize the lipidomic profile, the processed data were queried against the built-in lipid database in MS-DIAL, enabling comprehensive identification and annotation of lipid species.

**Figure 5:**
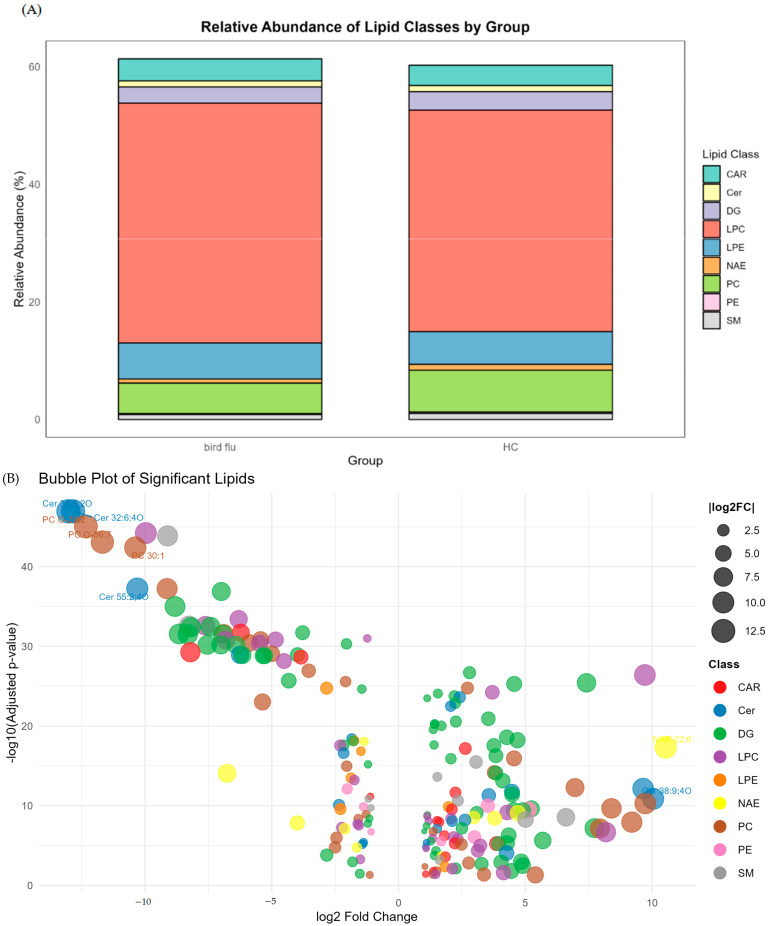
(**A**) Comparative analysis of lipid class composition between avian influenza-exposed and healthy control groups. (**B**) Bubble plot displays significantly dysregulated lipids between avian influenza-exposed and healthy control groups. Analyzed lipid classes include acylcarnitines (CAR), ceramides (Cer), diglycerides (DG), lysophosphatidylcholines (LPC), lysophosphatidylethanolamines (LPE), N-acylethanolamines (NAE), phosphatidylcholines (PC), phosphatidylethanolamines (PE), and sphingomyelins (SM).

Lipid class distribution was subsequently analyzed, and relative abundances of major lipid categories were quantified and visualized using a stacked bar chart (Figure 5A). The results revealed notable differences in lipid composition between the two groups. In both groups, lysophosphatidylcholine (LPC) was the most abundant lipid class, accounting for 40.83% in the exposed group and 37.68% in the control group, suggesting a general elevation of LPC species in individuals exposed to avian influenza. LPCs are known to play pro-inflammatory roles and act as signaling molecules in immune modulation and oxidative stress responses [26], indicating a potential link between viral antigen exposure and lipid-mediated inflammatory pathways. Other lipid classes showing moderate contributions included lysophosphatidylethanolamine (LPE) and diacylglycerol (DG). LPE levels were slightly elevated in the exposed group (6.10%) compared to controls (5.54%), while DGs were relatively higher in the control group (3.13%) than in the exposed group (2.75%). Interestingly, phosphatidylcholine (PC) abundance was markedly reduced in the exposed group (5.18%) compared to controls (7.13%). The observed increase in LPC alongside a decrease in PC is a common signature of altered phospholipid metabolism, suggesting an activation of phospholipase A2. PCs are essential for maintaining membrane integrity and serve as precursors for LPCs via phospholipase A2 activity [27], supporting the possibility of lipid disturbances associated with inflammatory or enzymatic processes. This lipidomic stacked bar chart indicated that occupational exposure to avian influenza may perturb lipid homeostasis, particularly through alterations in phospholipid metabolism and the generation of lipid-derived inflammatory mediators.

At the lipid species level, Figure 5B presents a bubble plot showing significantly dysregulated lipids (adjusted *p* < 0.05, fold change >2 or <0.5) between avian influenza-exposed and healthy control groups, with the most dramatically altered lipids labeled explicitly. Several ceramide species including Cer 32:6;4O, Cer 39:8;2O, and Cer 55:2;4O were among the most significantly downregulated lipids in the exposed group. Ceramides are bioactive sphingolipids involved in cell apoptosis, inflammation, and antiviral defense [28]. Their depletion may indicate increased catabolism or altered sphingolipid metabolism in response to chronic viral antigen exposure. Several sphingomyelin (SM) species, such as SM 27:1;2O and SM 21:2;2O, were also significantly reduced, suggesting disturbances in sphingolipid signaling and lipid composition. Notably, many ether-linked PC species (e.g., PC O-29:2, PC O-36:7) were dramatically down-regulated in the exposed group, suggesting increased oxidative stress or altered phospholipid metabolism, potentially reflective of membrane alterations. Conversely, LPC 20:2 showed marked up-regulation. LPEs such as LPE 18:3 and LPE O-16:1 also showed down-regulation, consistent with decreased availability or altered turnover of ethanolamine-linked lipids under stress or inflammation. These changes in LPC and LPE profiles point to a possible imbalance in phospholipid metabolism linked to viral exposure-induced immune activation and lipid metabolic alterations.

These lipidomic changes complement findings from metabolomic and pathway analyses, suggesting that avian influenza exposure is associated with alterations in lipid-related pathways that may influence immune processes, phospholipid homeostasis, and energy metabolism. Future targeted lipidomics studies are warranted to validate these signatures and explore their potential as early biomarkers of occupational viral exposure.

## 4. Limitation

Although we applied strict exclusion criteria (major infectious diseases, metabolic or autoimmune disorders, recent severe respiratory illnesses, pregnancy, lactation, heavy alcohol consumption, and heavy smoking) to minimize confounders, we acknowledge that this approach does not fully address broader socioeconomic or lifestyle differences such as dietary habits and physical activity levels. And it should be acknowledged that the presence of the virus in the workplace does not confirm that every participant inhaled a sufficiently infectious dose.

## 5. Conclusions

In this article, we employed an untargeted LC-MS-based metabolomics approach to investigate the serum metabolic alterations associated with occupational exposure to avian influenza. In particular, alanine, aspartate and glutamate metabolism and tryptophan metabolism were consistently identified as key dysregulated pathways, suggesting perturbations in nitrogen balance, mitochondrial energy production, and immune processes. Lipidomic profiling further revealed a substantial increase in LPC levels and a decrease in PC levels in the exposed group, indicating disturbances in phospholipid metabolism and pro-inflammatory lipid signaling triggered by viral antigen exposure. These findings provide valuable insights into the biochemical impact of avian influenza exposure and highlight candidate metabolic indicators of exposure-related physiological alterations, which may contribute to occupational health research and monitoring. Future studies with more detailed exposure histories are warranted to further elucidate the relationship between exposure duration and metabolic alterations.

## Figures and Tables

**Table 1:** The clinical characteristics of subjects recruited.

Clinical Characteristics	Bird Flu (n = 177)	Health Control (n = 59)	*p*-Value
Age (years)	44.94 ± 11.03	46.44 ± 9.22	0.307
Male/female	99/78	35/24	0.762

**Table 2:** Potential serum biomarkers in avian influenza exposed group.

ID	Metabolite Name	Fold Change	Regulation	*p* Value	Ontology
neg11330	9-HODE	0.06625251	↓	2.60 × 10^−25^	Lineolic acids and derivatives
neg12434	FA 18:2+2O	0.10958917	↓	2.87 × 10^−21^	Lineolic acids and derivatives
neg12973	9-HETE	0.02030262	↓	3.53 × 10^−44^	Hydroxyeicosatetraenoic acids
neg1780	Hypoxanthine	5.11259294	↑	3.82 × 10^−28^	Hypoxanthines
neg2115	Glutamine	2.7646163	↑	1.01 × 10^−66^	Alpha amino acids
neg21888	LPE 18:1	2.15101375	↑	0.03280666	1-acyl-sn-glycero-3-phosphoethanolamines
neg2332	Xanthine	2.25017518	↑	2.42 × 10^−12^	Xanthines
pos13087	gamma-Glutamylleucine	0.1499749	↓	2.11 × 10^−30^	Dipeptides
pos14218	FA 18:3+1O	0.09900637	↓	2.86 × 10^−20^	Medium-chain fatty acids
pos15560	Androstane-3,17-diol	0.04531475	↓	6.27 × 10^−16^	Androgens and derivatives
pos19515	LAUROYLCARNITINE	11.1916456	↑	4.82 × 10^−20^	Acyl carnitines
pos21869	Sphinganine 1-phosphate	0.46320622	↓	7.09 × 10^−31^	Phosphosphingolipids
pos24058	GLYCOCHENODEOXYCHOLATE	2.43241955	↑	7.22 × 10^−11^	Glycinated bile acids and derivatives
pos4781	Methionine	3.46770679	↑	1.06 × 10^−69^	Methionine and derivatives
pos6206	4-ACETAMIDOBUTANOATE	0.13766022	↓	1.66 × 10^−08^	Gamma amino acids and derivatives
pos6263	4-ureidobutanoic acid	2.28864439	↑	3.17 × 10^−39^	Gamma amino acids and derivatives
pos6904	3-Indoleacetic acid	6.39444246	↑	5.03 × 10^−15^	Indole-3-acetic acid derivatives

## Data Availability

Data are available in the Massive Data Repository (https://massive.ucsd.edu/, accessed on 1 October 2025) under the accession number MSV000098297.

## Supplementary Materials

The following supporting information can be downloaded at: https://www.mdpi.com/article/10.3390/metabo15100663/s1, Figure S1: Volcano plot of significantly altered metabolites in negative ion mode; Figure S2: MS/MS spectra of key metabolites: (A) Methionine, (B) Hypoxanthine and (C) Glycochenodeoxycholate. Table S1: Environmental Detection of Avian Influenza Virus in Workplaces of the Exposed Group.
