# Supplementary material for: Untargeted Metabolomics Reveals Distinct Serum Metabolic Profiles in Avian Influenza Occupational Exposure Populations
Source: Metabolites. 2025 Oct 11;15(10):663. doi: 10.3390/metabo15100663 (PMC12566296; doi:10.3390/metabo15100663)
Supplement: Supplementary file 1 [file metabolites-15-00663-s001.zip › Supplementary material.pdf]

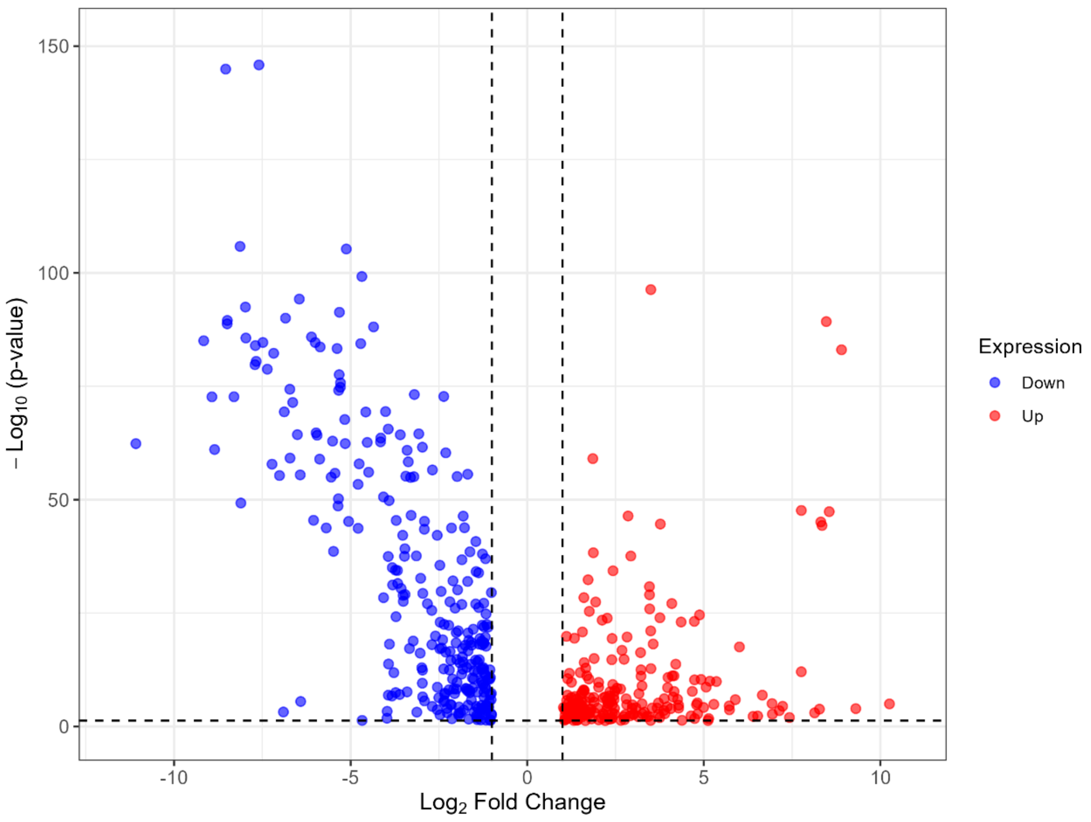

Figure S1. Volcano plot of significantly altered metabolites in negative ion mode.

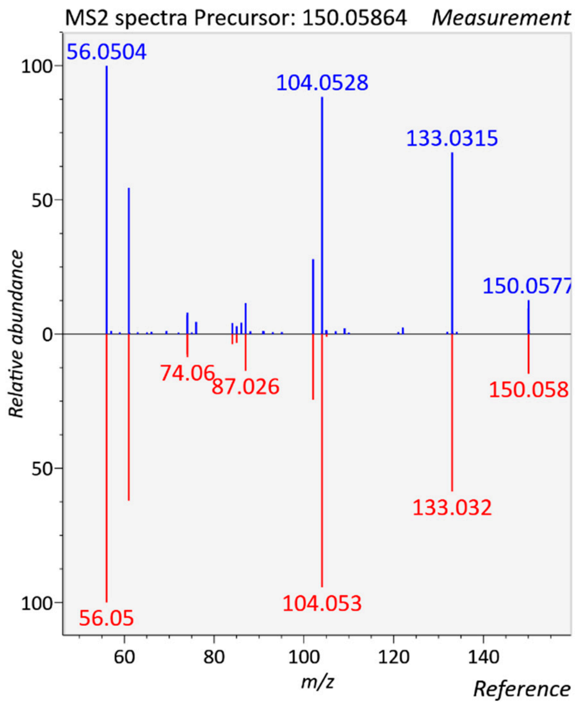

(A) Methionine

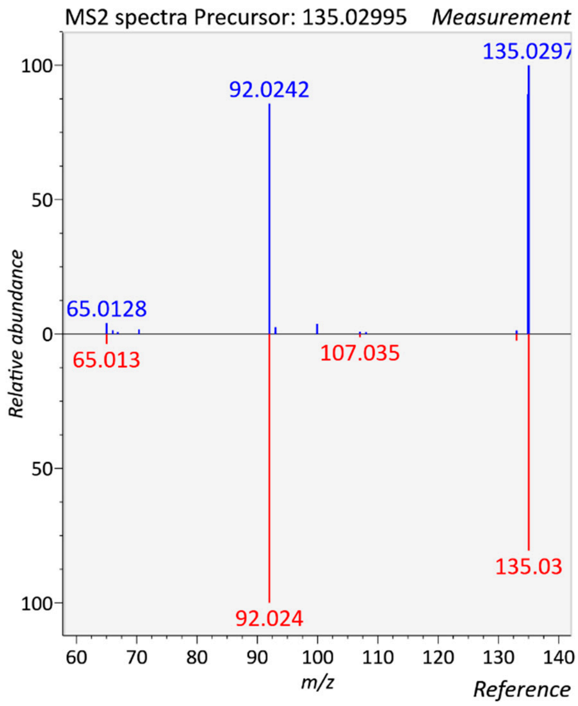

(B) Hypoxanthine

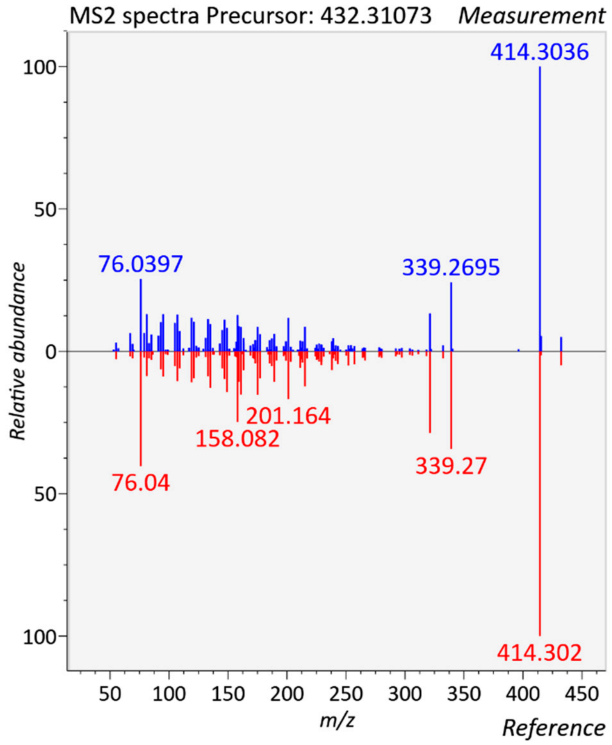

(C) Glycochenodeoxycholate

Figure S2. MS/MS spectra of key metabolites: (A) Methionine, (B) Hypoxanthine and (C) Glycochenodeoxycholate.
